# Genome-Wide Association Studies Identify Two Novel *BMP15* Mutations Responsible for an Atypical Hyperprolificacy Phenotype in Sheep

**DOI:** 10.1371/journal.pgen.1003482

**Published:** 2013-04-25

**Authors:** Julie Demars, Stéphane Fabre, Julien Sarry, Raffaella Rossetti, Hélène Gilbert, Luca Persani, Gwenola Tosser-Klopp, Philippe Mulsant, Zuzanna Nowak, Wioleta Drobik, Elzbieta Martyniuk, Loys Bodin

**Affiliations:** 1 INRA, UMR444 Laboratoire de Génétique Cellulaire, Castanet-Tolosan, France; 2 ENVT, UMR444, Laboratoire de Génétique Cellulaire, Castanet-Tolosan, France; 3 INRA, UMR85 Physiologie de la Reproduction et des Comportements, Nouzilly, France; 4 CNRS, UMR7247 Physiologie de la Reproduction et des Comportements, Nouzilly, France; 5 Université François Rabelais Tours, UMR Physiologie de la Reproduction et des Comportements, Nouzilly, France; 6 IFCE, UMR Physiologie de la Reproduction et des Comportements, Nouzilly, France; 7 Laboratorio di Ricerche Endocrino-Metaboliche, Istituto Auxologico Italiano, University of Milan, Milano, Italia; 8 Wydział Nauk o Zwierzętach, Katedra Genetyki i Ogólnej Hodowli Zwierząt, Warszawa, Polska; 9 INRA, UR0631 Station d'Amélioration Génétique des Animaux, Castanet-Tolosan, France; University of Bern, Switzerland

## Abstract

Some sheep breeds are naturally prolific, and they are very informative for the studies of reproductive genetics and physiology. Major genes increasing litter size (LS) and ovulation rate (OR) were suspected in the French Grivette and the Polish Olkuska sheep populations, respectively. To identify genetic variants responsible for the highly prolific phenotype in these two breeds, genome-wide association studies (GWAS) followed by complementary genetic and functional analyses were performed. Highly prolific ewes (cases) and normal prolific ewes (controls) from each breed were genotyped using the Illumina OvineSNP50 Genotyping Beadchip. In both populations, an X chromosome region, close to the *BMP15* gene, harbored clusters of markers with suggestive evidence of association at significance levels between 1E^−05^ and 1E^−07^. The *BMP15* candidate gene was then sequenced, and two novel non-conservative mutations called *FecX^Gr^* and *FecX^O^* were identified in the Grivette and Olkuska breeds, respectively. The two mutations were associated with the highly prolific phenotype (p*_FecX_^Gr^* = 5.98E^−06^ and p*_FecX_^O^* = 2.55E^−08^). Homozygous ewes for the mutated allele showed a significantly increased prolificacy (*FecX^Gr^/FecX^Gr^*, LS = 2.50±0.65 versus *FecX^+^/FecX^Gr^*, LS = 1.93±0.42, p<1E^−03^ and *FecX^O^/FecX^O^*, OR = 3.28±0.85 versus *FecX^+^/FecX^O^*, OR = 2.02±0.47, p<1E^−03^). Both mutations are located in very well conserved motifs of the protein and altered the BMP15 signaling activity *in vitro* using a BMP-responsive luciferase test in COV434 granulosa cells. Thus, we have identified two novel mutations in the *BMP15* gene associated with increased LS and OR. Notably, homozygous *FecX^Gr^/FecX^Gr^* Grivette and homozygous *FecX^O^/FecX^O^* Olkuska ewes are hyperprolific in striking contrast with the sterility exhibited by all other known homozygous *BMP15* mutations. Our results bring new insights into the key role played by the BMP15 protein in ovarian function and could contribute to a better understanding of the pathogenesis of women′s fertility disorders.

## Author Summary

Although causes altering reproductive function and leading to several fertility syndromes in women are various, a clear association between mutations in some members of the TGFβ family (BMP15 and GDF9) and abnormal ovarian phenotype has established the importance of these factors for normal female fertility. Some sheep breeds are naturally prolific since they carry major genes affecting ovulation rate and litter size. These species are therefore unique informative models for the study of reproductive genetics and physiology. Through a GWAS conducted in two sheep breeds using highly prolific ewes as cases and normal prolific ewes as controls, we identified an X chromosome locus close to the *BMP15* gene in both breeds. Two novel non-conservative *BMP15* mutations, one in each population, located in very well conserved domains of the protein were associated with the phenotype at heterozygous and homozygous status. Moreover, we showed that both mutations altered the BMP15 signaling activity, suggesting a novel kind of *BMP15* variant responsible for an atypical high prolificacy, in contrast to all other *BMP15* variants described so far. Our findings suggest an additional role of the BMP15 protein in folliculogenesis and could contribute to a better understanding of the pathogenesis of women′s fertility disorders.

## Introduction

There is strong evidence supporting the concept that oocyte plays a central role in follicle growth and developmental regulation [1], [2], [3]. It has been established that ovary-derived transforming growth factor-ß (TGFß) family members play an integral role during folliculogenesis. Indeed, the two paracrine factors Bone Morphogenetic Protein 15 (BMP15) and Growth and Differentiation Factor 9 (GDF9) stimulate follicle growth [4], [5], promote granulosa cell proliferation [6], [7], influence cell-survival signaling [8], [9] and modulate other growth factors and hormones [10], [11], [12].

The identification of *BMP15*, *GDF9* and Bone Morphogenetic Protein Receptor 1B (*BMPR1B*) gene mutations (Table 1) as the causal mechanism underlying either the highly prolific or infertile phenotypes of several sheep breeds in a dosage-sensitive manner highlighted the crucial role these genes play in ovarian function. The TGFß family member BMP15 was the first gene to be associated with prolificacy. All the known *BMP15* mutations (named *FecX^I,H,B,G,L or R^*) produce the same phenotype i.e. heterozygous ewes are highly prolific whereas homozygous females are infertile due to a blockade of follicular development at the primary stage [13], [14], [15], [16], [17]. A second member of the BMP pathway, BMPR1B, was later found to be associated with prolificacy. The genetic variant (*FecB^B^*) segregating in the Booroola Merinos breed presents an additive effect on ovulation rate (OR) and a partially dominant effect on litter size (LS) [18], [19], [20]. For *GDF9* (known as *FecG*), also member of the TGFß family, the two first described mutations present a phenotypic inheritance pattern similar to all *BMP15* variants [14], [21]. A third mutation in *GDF9* (*FecG^E^*) associated with prolificacy was identified recently in the Brazilian Santa Inês strain [22]. Interestingly, Silva *et al.* showed for the first time a novel phenotype since *FecG^E^* homozygous ewes are not sterile but exhibit a significant increase, compared to non mutated individuals, in their OR (2.22±0.12 vs. 1.22±0.11) and LS (1.78 vs. 1.13) [22].

**Table 1:** Genetics variants causing prolificacy phenotypes in sheep.

Gene	Mutation	Phenotype	Founder Breed	Reference
BMP15	*V299D*	Fecundity Inverdale, *FecX^I^*	Romney	[13]
	*Q291ter*	Fecundity Hanna, *FecX^H^*	Romney	[13]
	*S367I*	Fecundity Belclare, *FecX^B^*	Belclare	[14]
	*Q239ter*	Fecundity Galway, *FecX^G^*	Belclare and Cambridge	[14]
	*C321Y*	Fecundity Lacaune, *FecX^L^*	Lacaune	[15]
	*ΔP154S159*	Fecundity Rasa Aragonesa, *FecX^R^*	Rasa Aragonesa	[16], [17]
BMPR1B	*Q249R*	Fecundity Booroola, *FecB^B^*	Booroola Merino, Garole and Javanese	[18], [19], [20]
GDF9	*S395F*	Fecundity High Fertility, *FecG^H^*	Belclare, Cambridge	[14]
	*S427R*	Fecundity Thoka, *FecG^T^*	Icelandic	[21]
	*F345C*	Fecundity Embrapa, *FecG^E^*	Santa Inês	[22]

Similarly to sheep, a large number of mutations in the *GDF9* and *BMP15* genes have been described in women with fertility disorders. The identification of *GDF9* missense and nonsense mutations in premature ovarian failure (POF) patients [23], [24], [25] but also in mothers of dizygotic twins [26], [27], [28] suggests that altered GDF9 function is involved in ovarian dysfunction and polyovulatory phenotypes. Indeed, 3 missense variants *GDF9^P103S^*, *GDF9^P374L^* and *GDF9^R454C^* were significantly associated with an increased OR in women [27]. Critical roles of BMP15 in female fertility have also been demonstrated in women. The first *BMP15* mutation (*BMP15^Y235C^*) responsible for hypergonadotropic ovarian failure, due to ovarian dysgenesis, was detected in two sisters who inherited the variant from their father [29]. A number of other mutations and rare deletions in those 2 genes have been described in women with POF [24], [29], [30], [31], [32]. Recently, Hanevik *et al.* [33] confirmed results previously observed by Moron *et al.* [34] showing that a *BMP15* variant (*BMP15^−9G^*) was associated with ovarian hyperstimulation syndrome (OHSS), similar to effects observed in ewes with heterozygous *BMP15* mutations. Therefore, GDF9 and BMP15 are likely to alter OR and LS in women as well as in ewes. Interestingly, BMP15 regulates ovulation rate and female fertility in a species-specific manner, being crucial in humans and sheep and largely trivial in mice since loss-of-function of BMP15 results only in subfertility [35].

Although numerous mutations improving reproduction traits have been discovered in various sheep strains, the genetic variants have still to be identified for some other prolific sheep breeds. The French Grivette and the Polish Olkuska breeds present good maternal characteristics and abnormally high LS and OR, respectively. Moreover, both populations exhibit a very high variability of mean prolificacy among related females, suggesting the existence of an autosomal major gene for prolificacy segregating the same way as the *FecB^B^* mutation (reviewed in [36]). The *FecB^B^* or *FecX^I^* mutations do not account for the OR phenotype in Olkuska ewes [37]. This work aims at identifying genetic variants affecting LS and OR phenotypes in the French Grivette and Polish Olkuska sheep populations, respectively. A GWAS based on a case/control design followed by fine-mapping genetic analyses was performed. We identified 2 novel non-conservative mutations in the *BMP15* gene associated with an increase of LS and OR. Following the nomenclature used for previous fecundity genes, these 2 mutations were named *FecX^Gr^* in the Grivette population and *FecX^O^* in the Olkuska population. Noteworthy, homozygous ewes of both strains are highly prolific but not sterile as known so far. Our results bring new insights into the key role played by the BMP15 protein in ovarian function.

## Results

### Genetic association analyses

**Figure 1:**
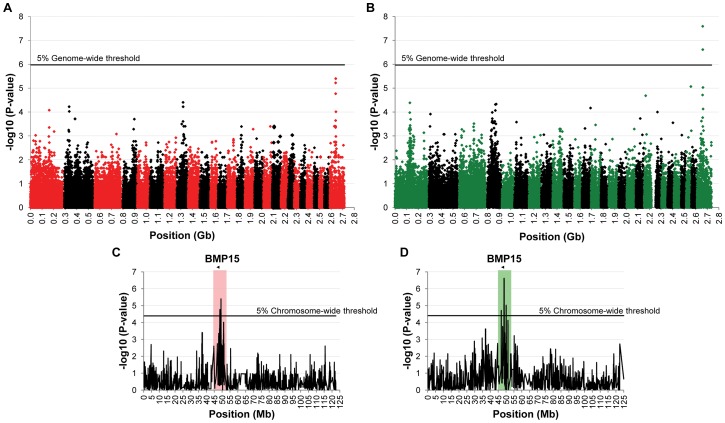
Genome-wide and chromosome-wide association results. (A) Genome-wide association results for litter size in the French Grivette sheep population. (B) Genome-wide association results for ovulation rate in the Polish Olkuska sheep population. Manhattan plots show the combined association signals (−log_10_(p)) on the y-axis versus SNPs position in the sheep genome on the x-axis and ordered by chromosome number (OARv2.0 available on http://www.livestockgenomics.csiro.au/sheep/ website). Black lines represent the 5% genome-wide threshold. Chromosomes are ordered from OAR1 to OAR26 and the X chromosome is the last one. (C) OARX chromosome-wide association results for litter size in the French Grivette sheep population. (D) OARX chromosome-wide association results for ovulation rate in the Polish Olkuska sheep population. Manhattan plots show the combined association signals (−log_10_(p)) on the y-axis versus SNPs position on the X chromosome (OARv2.0 available on http://www.livestockgenomics.csiro.au/sheep/ website). Black lines represent the 5% chromosome-wide threshold. Red and green boxes pinpoint locus where significant association results are obtained for several markers in the Grivette and Olkuska populations, respectively. The location of the *BMP15* gene is mentioned by an arrow.

To identify loci associated with highly prolific phenotype in the French Grivette and the Polish Olkuska sheep breeds, a GWAS comparing allele frequencies between highly prolific ewes (cases) versus normally prolific ewes (controls) was conducted as outlined in Materials and Methods and Table S1. Genotype data from the Illumina OvineSNP50 Genotyping Beadchip was obtained for 39 ewes (28 cases *vs.* 11 controls) and 63 ewes (29 cases *vs.* 34 controls) from the Grivette and the Olkuska populations, respectively. No significant genome-wide association after Bonferroni correction was found in the French Grivette breed although an association was suggested for a cluster of markers located on the X chromosome (Figure 1A). Indeed, 5 OARX SNPs, in a region close to *BMP15* gene, present evidence of association at chromosome-wide level as shown on Figure 1C and in Table 2. For the Polish Olkuska breed, 2 and 4 markers located on OARX close to the *BMP15* gene are significantly associated with OR at genome-wide and chromosome-wide levels, respectively (Figure 1B, 1D and Table 2).

**Table 2:** Markers significantly associated with prolificacy.

Sheep Population	SNP	Chromosome	a Position (bp)	b MAF	c p _Unadjusted_	d p _Genome-wide corrected_	e p _Chromosome-wide corrected_
Grivette	OARX_53514671	OARX	48531006	0.12	1.70E^−05^	8.03E^−01^	2.16E^−02^
Grivette	s53538	OARX	48569907	0.12	1.70E^−05^	8.03E^−01^	2.16E^−02^
Grivette	OARX_53305527	OARX	48779080	0.12	1.70E^−05^	8.03E^−01^	2.16E^−02^
Grivette	OARX_53225720	OARX	48852270	0.03	1.70E^−05^	8.03E^−01^	2.16E^−02^
Grivette	OARX_52851975	OARX	49267300	0.15	3.96E^−06^	1.87E^−01^	5.04E^−03^
Olkuska	OARX_54412853	OARX	46954696	0.44	1.92E^−05^	8.93E^−01^	2.45E^−02^
Olkuska	s56924	OARX	48706133	0.48	2.41E^−07^	1.12E^−02^	3.06E^−04^
Olkuska	OARX_53305527	OARX	48779080	0.48	2.41E^−07^	1.12E^−02^	3.06E^−04^
Olkuska	OARX_51960372	OARX	50080732	0.20	9.34E^−06^	4.34E^−01^	1.19E^−02^

a : Location of markers are based on the OARv2.0 assembly available on http://www.livestockgenomics.csiro.au/sheep/ website.

b : MAF for Minor Allele Frequency.

c : p _Unadjusted_ corresponds to exact p for the Fisher's test.

d : p _Genome-wide corrected_ corresponds to p after genome-wide Bonferroni correction.

e : p _Chromosome-wide corrected_ corresponds to p after chromosome-wide Bonferroni correction.

**Figure 2:**
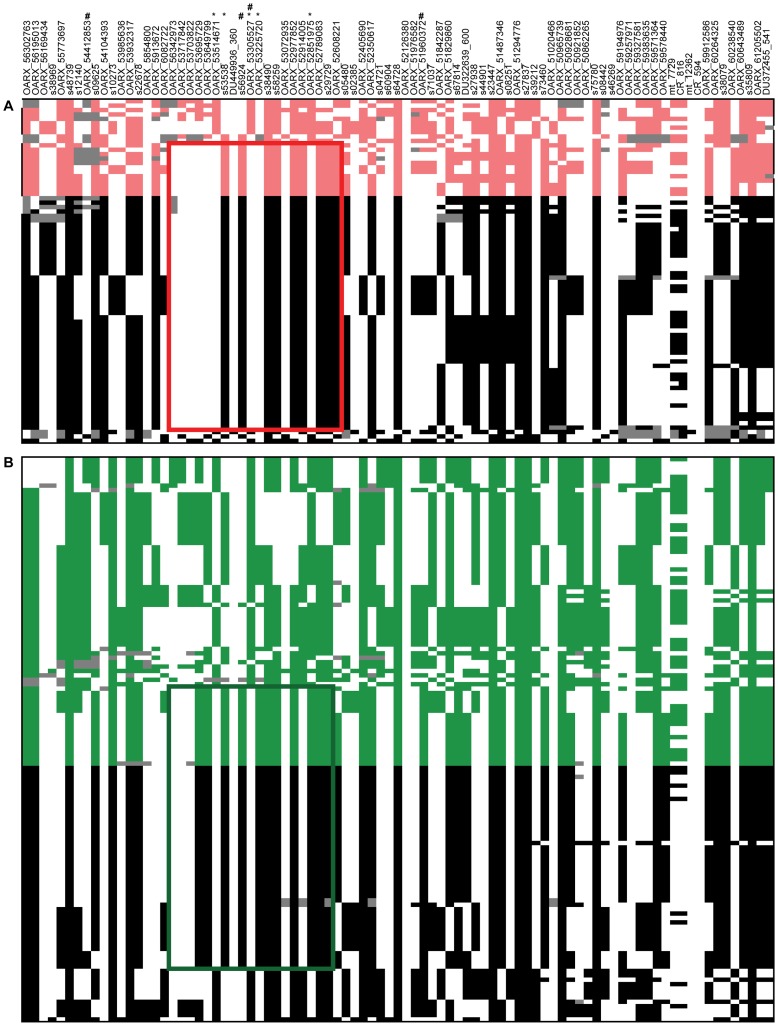
Clusterization of haplotypes reconstructed at the OARX locus. (A) Haplotypes determined in the French Grivette sheep population. (B) Haplotypes determined in the Polish Olkuska sheep population. 87 markers located in the interest OARX region (45 Mb–55 Mb) were selected to reconstruct haplotypes. Each column represents one SNP and each line represents one haplotype. For one marker (*i*) allele 1 is in red (Grivette) or green (Olkuska) in controls, respectively or black in cases, (*ii*) allele 2 is in white when the phase was unambiguous and (*iii*) dark grey colour represents unphased SNP. Haplotypes were ordered to distinguish controls versus cases and clusterized to classify similar clades of haplotypes. Markers with evidence of association at significance levels are marked with a star or a hash in French Grivette and Polish Olkuska sheep populations, respectively. In both breeds, the specific haplotype preferentially selected in highly prolific ewes (cases) is symbolized by red (Grivette) and green (Olkuska) boxes. The *BMP15* gene (48140251 bp–48146740 bp) is located between markers named OARX_6082722 and OARX_56342973.

To further characterize the X chromosome locus, we selected 87 markers spanning the 10 Mb region (from 45 Mb to 55 Mb according to the OARv2.0 assembly available on (http://www.livestockgenomics.csiro.au/sheep/ website) and we determined the most likely linkage phase for each individual. After haplotype clusterization for each population, we identified a specific segment that was more frequent in highly prolific ewes than in control individuals as shown on Figure 2A and 2B. In the French Grivette population, a significant association was found for the 1.3 Mb (48240883 bp–49526964 bp) haplotype containing 20 markers (Fr_cases_ = 0.95 *vs.* Fr_controls_ = 0.54, p_corrected_ = 9.90E^−03^) (Figure 2A). Similarly, a different haplotype but located in the same region (48240883 bp–49381143 bp) was shown significantly associated with the OR phenotype in the Polish Olkuska breed (Fr_cases_ = 0.76 *vs.* Fr_controls_ = 0.27, _corrected_ = 9.90E^−04^) (Figure 2B).

### Characterization of mutations

The very close location of the identified region to the *BMP15* gene (48140251 bp–48146740 bp) and the crucial role of BMP15 in ovarian function prompted us to consider *BMP15* as a positional and functional candidate gene. The *BMP15* coding sequence was obtained for all the individuals genotyped in GWAS for both sheep breeds.

**Figure 3:**
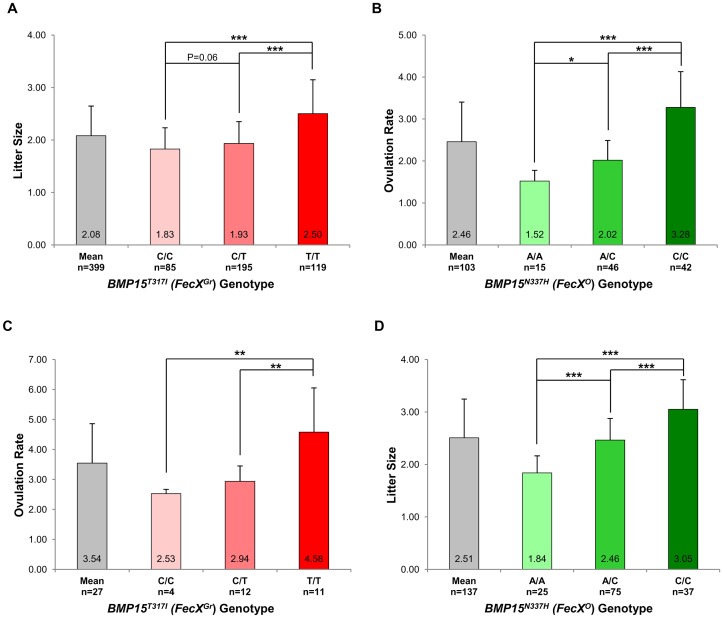
Genotypic distributions of ***FecX^Gr^*** **and** ***FecX^O^*** **mutations.** (A) Genotypic distribution of the *BMP15^T317I^ (FecX^Gr^)* in the French Grivette sheep population for the litter size phenotype. (B) Genotypic distribution of the *BMP15^N337H^ (FecX^O^)* in the Polish Olkuska sheep population for the ovulation rate phenotype. (C) Genotypic distribution of the *BMP15^T317I^ (FecX^Gr^)* in the French Grivette sheep population for the ovulation rate phenotype. (D) Genotypic distribution of the *BMP15^N337H^ (FecX^O^)* in the Polish Olkuska sheep population for the litter size phenotype. The means LS or OR in breeds are firstly presented then ewes were ordered according to their genotype at the mutation of interest. Means±SD for prolificacy were calculated for the 3 groups of genotype and are noted into each histogram bar. Number of ewes counted per group of genotype is mentioned (n). Pairwise statistical comparisons using a one-way ANOVA test between means of genotype's clades were performed and results of statistic test are symbolized by stars. p: * = p<5E^−02^; ** = p<1E^−02^ and *** = p<1E^−03^.

In the French Grivette population, 3 polymorphisms were identified: the already known c.28–30delCTT and c.747C>T and a new c.950C>T leading to the ΔL10 deletion, the P249P synonymous substitution and a non-conservative (T317I) substitution (Figure S1A), respectively as shown in Table 3. The distribution of the *BMP15^T317I^* mutation strongly suggested its association with increased litter size since 26 out of the 28 highly prolific ewes were homozygous for the T mutated allele (Table 3). To validate this hypothesis, the *BMP15^T317I^* genotype was added to the GWAS and a significant chromosome-wide association was found (p_unadjusted_ = 5.98E^−06^, p_Genome-wide corrected_ = 2.83E^−01^ and p_Chromosome-wide corrected_ = 7.61E^−03^) (Figure S2A). Preliminary genetic results were confirmed *via* the genotyping of 360 additional ewes randomly chosen in 9 flocks and then litter size means between the 3 genotypes at *BMP15^T317I^* (C/C *vs.* C/T *vs.* T/T) were compared as shown on Figure 3A. Litter size of individuals with the T/T genotype (n = 119, LS = 2.50±0.65) was significantly higher than litter sizes of ewes with the C/C (n = 85, LS = 1.83±0.41) or the C/T (n = 195, LS = 1.93±0.42) genotypes (p<1E^−03^). However, there was no difference between LS from C/T and C/C genotypes although a trend was observed (p = 0.06). These results were confirmed on the ovulation rate measured for a few ewes (n = 27) (Figure 3C). Additionally, 6 kb of the 5′ regulatory region of the *BMP15* gene were sequenced in 2 extreme ewes (homozygous C/C, LS = 1.17 and homozygous T/T, LS = 4.33) and no polymorphism was identified, therefore reinforcing the role of the *BMP15^T317I^* substitution.

**Table 3:** BMP15 genetic variants identified in the Grivette and the Olkuska populations.

Variant	Base change	Coding Base (bp)^a^	Coding residue (bp)^b^	Amino Acid Change	Genotype					
*Grivette*					*Controls*			*Cases*		
*ΔL10*	ΔCTT	28–30	10	Deletion Leu	+/+ (0)	+/− (5)	−/− (6)	+/+ (0)	+/− (1)	−/− (27)
*P249P*	C>T	747	249	Unchanged Pro	C/C (0)	C/T (2)	T/T (9)	C/C (0)	C/T (0)	T/T (28)
***BMP15^T317I^*** **(** ***FecX^Gr^*** **)**	**C>T**	**950**	**317**	**Thr>Ile**	**C/C (2)**	**C/T (6)**	**T/T (3)**	**C/C (0)**	**C/T (2)**	**T/T (26)**
*Olkuska*										
*ΔL10*	ΔCTT	28–30	10	Deletion Leu	+/+ (28)	+/− (7)	−/− (0)	+/+ (29)	+/− (0)	−/− (0)
*A77A*	T>G	231	77	Unchanged Ala	T/T (8)	T/G(27)	G/G (0)	T/T (3)	T/G (26)	G/G (0)
*P101A*	G>C	301	101	Pro>Ala	G/G (8)	G/C (27)	C/C (0)	G/G (3)	G/C (26)	C/C (0)
*L110L*	C>T	330	110	Unchanged Leu	C/C (3)	C/T (32)	T/T (0)	C/C (5)	C/T (24)	T/T (0)
*V135G*	T>G	404	135	Val>Gly	T/T (34)	T/G (1)	G/G (0)	T/T (29)	T/G (0)	G/G (0)
***BMP15^N337H^*** **(** ***FecX^O^*** **)**	**A>C**	**1009**	**337**	**Asn>His**	**A/A (16)**	**A/C (19)**	**C/C (0)**	**A/A (1)**	**A/C (9)**	**C/C (19)**

a : Position of the coding base in the *Ovis aries* bone morphogenetic protein 15 (BMP15) mRNA (GenBank accession number NM_001114767.1).

b : Position of the coding residue in the *Ovis aries* bone morphogenetic protein 15 (BMP15) protein (GenBank accession number AAF81688.1).

Numbers in brackets indicate number of ewes carrying the genotype.

The variants in bold are associated with high prolificacy.

A similar strategy was used for the Polish Olkuska breed, and 6 polymorphisms were identified including 5 new ones. Among them, c.231C>T and c.330C>T led to synonymous substitutions (A77A and L110L), and c.301G>C, c.404T>G and c.1009A>C cause P101A, V135G and N337H non-conservative substitutions (Figure S1B), respectively (Table 3). Only the *BMP15^N337H^* mutation was shown to be more frequent in cases than in controls (0.81 *vs.* 0.26) (Table 3) and was significantly associated to OR at a genome-wide level (p_unadjusted_ = 2.55E^−08^, p_Genome-wide corrected_ = 1.18E^−03^) (Figure S2B). Analysis of the genotypic distribution of the *BMP15^N337H^* polymorphism in additional Olkuska ewes confirmed the key role of the mutation on OR. Indeed, the 3 groups of genotype showed statistically different ovulation rate means (A/A, OR = 1.52±0.26 *vs.* A/C, OR = 2.02±0.47, p<5E^−02^; A/A, OR = 1.52±0.26 *vs.* C/C, OR = 3.28±0.85, p<1E^−03^ and A/C, OR = 2.02±0.47 *vs.* C/C, OR = 3.28±0.85, p<1E^−03^) as shown on Figure 3B. These results were validated on the litter size phenotype (Figure 3D). Polymorphisms were identified in the 6 kb 5′ regulatory region of the BMP15 gene of 4 extreme animals but none of them seemed to be associated with the high prolificacy emphasizing the interest of the *BMP15^N337H^* substitution (Table S2).

Altogether, this data suggests that the non-conservative *BMP15^T317I^* and *BMP15^N337H^* mutations, called *FecX^Gr^* and *FecX^O^*, respectively, are causal mutations. Both mutations are associated with increased litter size and ovulation rate in the French Grivette and Polish Olkuska sheep populations. Interestingly, homozygous ewes are highly prolific and not sterile, a phenotype discordant from all BMP15 mutations described so far in sheep.

### Functional effects of mutations

**Figure 4:**
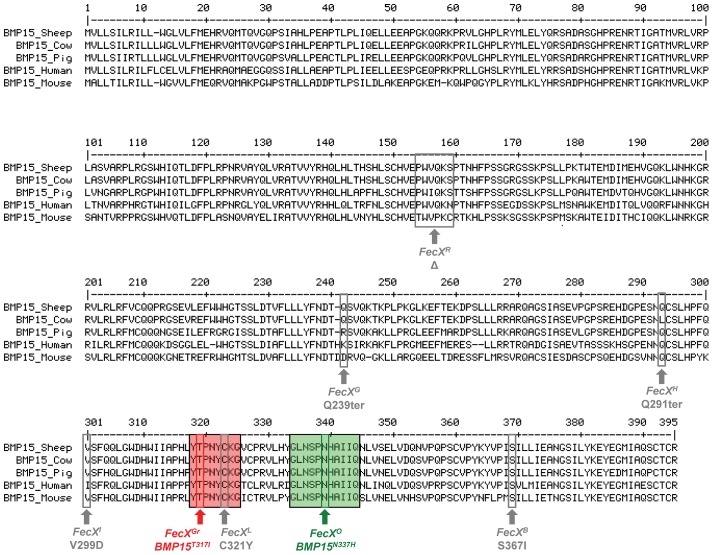
***BMP15*** **multi-species sequences alignment and position of sheep mutations.** The BMP15 protein sequences from *Ovis aries* (GenBank AAF81688.1), *Bos taurus* (GenBank DAA12832.1), *Sus scrofa* (GenBank NP_001005155.1), *Mus musculus* (GenBank NP_033887.1) and *Homo sapiens* (GeneBank NP_005439.2) were aligned and compared. Grey boxes and arrows represent known mutations as reviewed in Otsuka et al. [12]. *FecX^Gr^* and *FecX^O^* mutations are in red or green frames, respectively, themselves into bigger frames symbolizing conserved protein motifs.

Both *FecX^Gr^* and *FecX^O^* mutations are closely located into two very well conserved domains of the sheep, cow, pig, human and mouse BMP15 proteins (Figure 4). Impacts of *BMP15^T317I^* and *BMP15^N337H^* variants on the intrinsic properties of the BMP15 protein were estimated by comparing hydrophobicity, polarity and molecular weight between mutated and wild type BMP15 proteins. *FecX^Gr^*, which corresponds to a substitution of a threonine to an isoleucine, clearly affected the hydrophobicity of the protein while *FecX^O^* altered the polarity and the molecular weight of the protein by replacing an asparagine to a histidine as shown on Figure S3.

**Figure 5:**
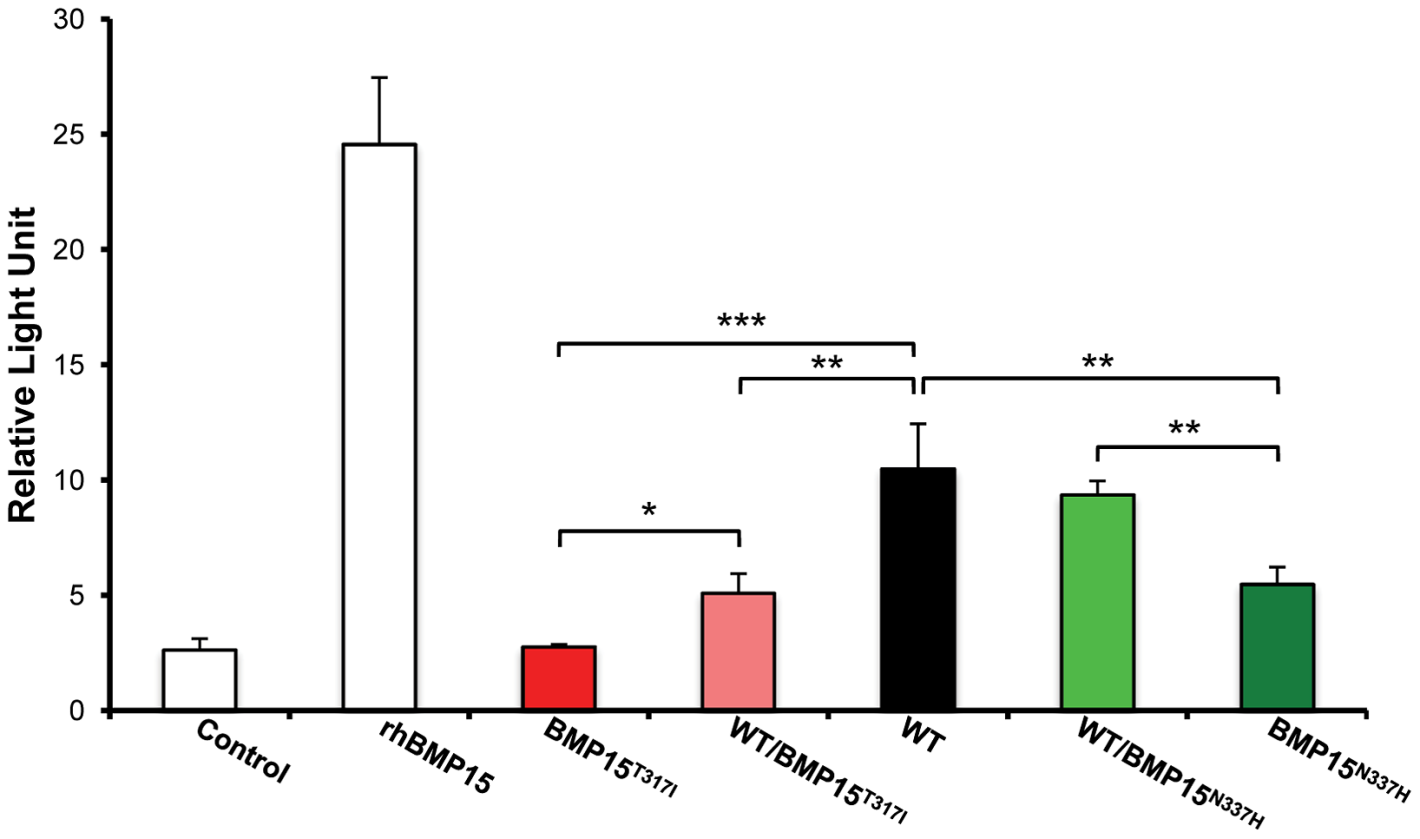
Functional effects of ***FecX^Gr^*** **and** ***FecX^O^*** **mutations on the BMP15 activity.** *In vitro* reporter luciferase assay from COV434 granulosa cells transiently transfected with empty vector +/− 100 ng of recombinant human BMP15 (Control +/− rhBMP15) or wild-type human BMP15 expressing vector (WT) or the 2 different BMP15 variant vectors (*BMP15^T317I^ (FecX^Gr^)*; *BMP15^N337H^ (FecX^O^)*) obtained by directed-mutagenesis. Results are expressed as Means±SD of the relative light unit (RLU) from 3 independent experiments in triplicate for each condition. Pairwise statistical comparisons using a one-way ANOVA test between means were performed and results of statistic test are symbolized by stars: * = p<5E^−02^; ** = p<1E^−02^ and *** = p<1E^−03^.

To evaluate the effects and then the causality of these two naturally occurring mutations on the BMP15 signaling activity, each mutated allele was introduced by targeted mutagenesis in the human BMP15 expressing plasmid. The functional assay was realized *in vitro* using a human granulosa-derived COV434 cell line stably expressing a BMP-responsive element luciferase reporter construct, as previously described [32]. The quantification of the luciferase activity after transient transfection was compared between plasmids expressing the wild-type allele, the mutated allele and a mix of both, mimicking the heterozygous status (Figure 5). As positive controls, COV434 cells expressing the luciferase reporter gene showed a significant activation of the BMP signaling pathway when stimulated with 100 ng of the exogenous recombinant human BMP15 (p<1E^−03^), or when transiently transfected with the wild-type human BMP15 expressing construct (p<1E^−03^). In comparison to the wild-type, the *BMP15^T317I^* mutation totally abolished the BMP15 signaling activity. When the *BMP15^T317I^* plasmid was cotransfected with an equal amount of the wild-type form, the impaired luciferase activity was partially rescued. Concerning the *BMP15^N337H^* mutation, it altered also the BMP15 signaling by half-reducing its activity (p<1E^−02^) and this was totally rescued by the *BMP15^T317I/WT^* cotransfection. This data shows that the two novel mutations drastically affect the signaling pathway of BMP15 and thus, supports their causal role into the highly prolific phenotype observed in the French Grivette and Polish Olkuska sheep populations.

## Discussion

Some sheep breeds are naturally highly prolific and they are very informative for the studies of reproductive genetics and physiology. Genetic studies have indicated that LS and OR can be genetically determined by the action of single genes with a major effect, named fecundity (Fec) genes [38]. All the three Fec genes discovered so far belong to the TGFß superfamily: *BMPR1B* gene (*FecB*) located on OAR6 [18], [19], [20], *GDF9* gene (*FecG*) located on OAR5 [14], [21], [22] and *BMP15* gene (*FecX*) located on the X chromosome [13], [14], [15], [16], [17].

### Effect of mutations on the prolificacy phenotype

Segregation of major genes increasing LS and OR was suspected in the French Grivette and the Polish Olkuska sheep flocks, respectively. In the Olkuska breed, a previous study had rejected the hypothesis of the segregation of 2 already known mutations, *FecX^I^* and *FecB^B^* [37]. Due to an atypical highly prolific phenotype with no sterile individual and no presence of streak ovaries in both breeds, *a priori* excluding the *FecX* gene, a GWAS based on a case/control design was performed to identify a causal locus in each population. A significant association was found for a cluster of SNPs located on the X chromosome close to the *BMP15* gene suggesting its possible involvement. We identified 2 novel non-conservative mutations in the *BMP15* gene called *FecX^Gr^* and *FecX^O^* for the Grivette and the Olkuska variants, respectively. However, due to the specific genetic background of each breed as well as the difference of measured traits (OR vs LS), at this stage, the effect of the mutations appears to be slightly different for Olkuska and Grivette. Noteworthy, *FecX^Gr^* and *FecX^O^* led to highly prolific ewes at the homozygous status in contradiction with other *BMP15* variants described so far. Indeed, all the *FecX* variants (6 different mutated alleles) were associated with an increased LS/OR in heterozygous animals but sterility in homozygous animals [13], [14], [15], [16]. Then, it was unexpected to identify mutations in the *BMP15* gene exclusively responsible for a high prolificacy phenotype. Nevertheless, an identical phenotypic effect has been recently shown for a new *GDF9* allele called *FecG^E^* in the Brazilian Santa Ines sheep breed. Silva *et al.*, [22] described for the first time, a polymorphism in the *GDF9* gene that increases the ovulation rate (82%) and prolificacy (58%) in fertile homozygous ewes. Therefore, our two novel mutations (*FecX^Gr^* and *FecX^O^*) as well as *FecG^E^* are associated with a prolific phenotype in homozygous ewes.

### Effect of mutations on the intrinsic BMP15 protein properties

Both *FecX^Gr^* and *FecX^O^* mutations are closely located into two very well conserved domains of the sheep, cow, pig, human and mouse BMP15 proteins. The BMP15 protein belonging to the TGFβ family is translated as a pre-proprotein which consists of a signal peptide, a large proregion and a mature peptide (reviewed in [39]). In sheep, 2 (*FecX^R^* [16], [17] and *FecX^G^* [14]) and 6 (*FecX^H^* [14], *FecX^I^* [13], *FecX^L^* [15], *FecX^B^* [14], *FecX^Gr^ and FecX^O^*) *BMP15* mutations have been found in the proregion and the mature protein, respectively. The effect of sheep variants seems tightly related to the kind of mutations described. Indeed, 3 out of the 8 mutations identified so far are aminoacids deletion (*FecX^R^* [16], [17]) or premature stop codon (*FecX^G^* and *FecX^H^*) [14] in the *BMP15* sequence impairing consequently the production of the BMP15 active form. Our mutations are located in the mature BMP15 protein, on both sides of the *FecX^L^* mutation. A putative misfolding inhibiting the maturation/production of the BMP15 protein was suspected in the case of the *FecX^L^* mutation which leads to a substitution of a cysteine with a tyrosine in one of the six conserved cysteines involved in the characteristic folding of the TGFβ factors [15]. The identified *FecX^Gr^* and *FecX^O^* mutations convert threonine to isoleucine and asparagine to histidine in the French Grivette and the Polish Olkuska sheep populations, respectively. These two mutations clearly affect the intrinsic properties of the BMP15 protein since they correspond to substitutions of polar aminoacids by nonpolar and basic aminoacids suspected to modify consequently its three dimensional structure.

### Effect of mutations on the BMP15 signaling pathway

Functionally, both *FecX^Gr^* and *FecX^O^* mutations clearly impaired the BMP15 signaling as shown by our *in vitro* test in COV434 cells. Indeed, *BMP15^T317I^* induced a complete inhibition of BMP15 *in vitro* activity whereas the *BMP15^N337H^* only partially disturbed BMP15 signaling. The partial loss-of-function observed for the *BMP15^N337H^* mutation fits well with the hyperprolific phenotype in homozygous Olkuska sheep following the dosage-sensitive hypothesis of BMP15 action on OR [13], [40]. *In vitro* effect of *FecX^Gr^* on the BMP15 pathway is similar to the *FecX^L^* variant [32] although prolificacy phenotypes of homozygous ewes are opposite i.e. *FecX^L^*/*FecX^L^* animals are sterile whereas *FecX^Gr^*/*FecX^Gr^* individuals are highly prolific. Such functional discrepancy has been already observed when comparing *FecX^L^* with other known non-conservative substitutions in the BMP15 mature peptide such as *FecX^I^* and *FecX^B^*. Indeed, functional analyses of *FecX^I^* and *FecX^B^* mutations indicated that neither V299D nor S367I substitutions are able to alter the production, processing, homodimerization, or biological activity of the BMP15 protein [41] while homozygous animals are sterile. The underlying mechanism occurs through the heterodimerization of BMP15 with the closely related oocyte-derived GDF9 protein [41], [42], [43] and their cooperative effect on granulosa cells [44], [45], [46]. Although the *FecX^Gr^* mutation totally impaired the BMP15 direct signaling, it would still maintain a biological activity through its interaction with GDF9 suggesting a normal folding of the BMP15 protein in contrast to the *FecX^L^* variant [15]. Furthermore, we hypothesized that the *BMP15^T317I^* null mutation doesn't prevent the heterodimerization process with GDF9 compared to the *FecX^I^* variant for which the heterodimer signaling pathway has been implicated [41], [42], [43]. To better understand molecular effects of both *FecX^Gr^* and *FecX^O^* mutations, independent actions of BMP15 and GDF9 as well as their synergic role remains to be determined.

### Model of the ovulation quota control by the various Fecundity mutations

**Figure 6:**
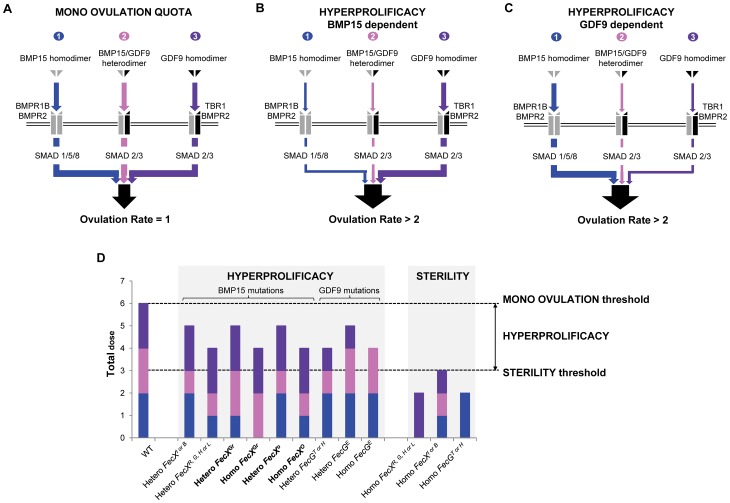
Hypothesized overview of ovulation quota control by the various Fecundity mutations. (A) The mono ovulation quota is tightly controlled by the integrative action of *i)* BMP15 homodimer through BMPR1B, BMPR2 and SMAD1/5/8, *ii)* BMP15/GDF9 heterodimer through SMAD2/3 and *iii)* GDF9 homodimer through TBR1, BMPR2 and SMAD2/3. (B) Increased of ovulation rate due to *FecX* mutations. This drawing represents an overview of hyperprolificacy dependent of BMP15 variants. Heterozygous or homozygous *FecX* mutations leading to an increased OR affect either the signaling pathways of BMP15 homodimer, BMP15/GDF9 heterodimer or both whereas the GDF9 homodimer signaling pathway remains stable. (C) Increased of ovulation rate due to *FecG* mutations. This drawing represents an overview of hyperprolificacy dependent of GDF9 variants. Heterozygous or homozygous *FecG* mutations leading to an increased OR impair either the signaling pathways of GDF9 homodimer, BMP15/GDF9 heterodimer or both whereas the BMP15 homodimer signaling pathway remains stable. (D) Dose sensitive effect of each *FecX* and *FecG* mutations on the BMP15, GDF9 homodimers and BMP15/GDF9 heterodimer signaling pathways. Based on the various *in vitro* tests performed, we assigned a dose (n = 0, 1 or 2) to each signaling pathway in an attempt to explain the prolificacy phenotype observed for all the *FecX* and *FecG* mutations. When the ovulation rate is normal ( = 1), i.e. the WT situation, 2 doses of BMP15 homodimer (blue), GDF9 homodimer (purple) and BMP15/GDF9 heterodimer (pink) were considered. As example, in the case of the *FecX^Gr^* mutation where the BMP15 homodimer signaling pathway seemed clearly affected at homozygous and heterozygous status (as shown on Figure 5), we assumed that the BMP15/GDF9 heterodimer signaling pathway remained totally active.

Based on the current knowledge, the mono ovulation quota seems to be tightly controlled by the triple action of *i)* BMP15 homodimer and its signaling pathway through BMPR1B, BMPR2 and SMAD1/5/8 [47], *ii)* BMP15/GDF9 heterodimer signaling also through SMAD2/3 [46] and *iii)* GDF9 homodimer signaling through TBR1, BMPR2 and SMAD2/3 [48], [49] (Figure 6A). However, the integration of these 3 pathways under a threshold activity precociously blocks the folliculogenesis and leads to sterility. This could be illustrated by the *FecX^L, H, G, R^* effect directly abolishing the BMP15 production [13], [14], [15], [16], [17] thus impairing at least BMP15 homodimer and BMP15/GDF9 heterodimer signaling. Moreover, when the *FecX^I^* or *FecX^B^* mutants are coexpressed with normal GDF9, the secretion of both BMP15 and GDF9 is significantly reduced [42]. Although effects of *FecG^H^* and *FecG^T^* variants have never been tested *in vitro*, it is strongly thought that GDF9 loss-of-function mutations might alter both GDF9 homodimer and BMP15/GDF9 heterodimer pathways leading to infertile animals. When the activity of the combined action of the 3 pathways is in between the sterility threshold and the normal level, i.e. heterozygous status, OR and LS are increased through modulation of granulosa cells proliferation and gonadotropin sensitivity [38], [40]. It is likely that Fecundity mutations resulting in hyperprolificacy without sterility might affect either totally the BMP15, GDF9 homodimers signaling pathways or partially the homodimer and heterodimer signaling pathways (Figure 6B and 6C). Our *FecX^Gr^* and *FecX^O^* as well as *FecG^E^* mutations support this hypothesis. Indeed, the *FecX^O^* mutation would mainly have an effect on the BMP15/GDF9 heterodimer signaling *via* the heterodimerization process whereas the *FecX^Gr^* mutation clearly impairs the BMP15 homodimer pathway through the BMP15 homodimerization or the binding to its receptor (Figure 6D). This assumption is close to the Booroola (*FecB^B^*) homozygous hyperprolific phenotype where a loss-of-function mutation altered the BMP15 receptor BMPR1B [50] and then suggested that only the BMP15 homodimer signaling pathway was affected. Concerning the *FecX^Gr^* mutation for which the abolishment of the Smad 1/5/8 pathway was observed, it is also possible that another BMP15 signaling pathway, the TAK1 pathway, remains functional maintaining a biological BMP15 activity [46]. This assumption would explain why homozygous *FecX^Gr^*/*FecX^Gr^* ewes are not sterile.

### Comparison between human and sheep BMP15 mutations

The BMP15 protein has also been described in women to play critical roles in female fertility. Indeed, a large number of mutations in the *BMP15* gene have been identified in women with POF [24],[29],[30],[31] and in patients with OHSS [33], [34] but none are in common between women and sheep. Interestingly, all the human *BMP15* mutations described so far and involved in the POF syndrome were found at a heterozygous status. Therefore, heterozygous carriers exhibit an ovarian phenotype [29] similar to sterile homozygous *FecX* ewes which present an early folliculogenesis arrest, ovarian dysgenesis and streak ovaries [13], [15]. In contrary, heterozygous and homozygous ewes carrying *FecX^Gr^* or *FecX^O^* mutations show an increase of LS and OR with normal and functional ovaries. Our data suggest that these two variants are novel kind of *BMP15* mutations responsible for a new prolificacy phenotype as it was already shown in the case of the *FecG^E^* allele [22]. Noteworthy, BMP15 was recently involved in women suffering of OHSS since a *BMP15* variant (*BMP15^−9G^*) was associated with this disorder [33], [34], similarly to reported effects of heterozygous *BMP15* mutations in sheep. Although the connection between the *BMP15^−9G^* SNP and OHSS was mainly attributable to the considerably high number of heterozygous patients, a few women cases *BMP15^−9G^*/*BMP15^−9G^* homozygous were also identified and contribute to the significant signal obtained [33]. Therefore, the atypical phenotypic effect (high prolificacy) observed in ewes carrying *FecX^Gr^* or *FecX^O^* mutations seems somewhat comparable to the OHSS in women at both heterozygous and homozygous status. Further studies need to be conducted to characterize the physiological ovarian phenotype of the highly prolific Grivette and Olkuska individuals.

In summary, our results bring new insights into the key role played by the BMP15 protein in ovarian function. The *BMP15* gene is almost the only gene identified to date whose mutations in mammals as sheep and human result in impaired early folliculogenesis and POF as well as excessive ovulation rate and OHSS. Although the BMP15 biological effects might be specie-specific, parallels between phenotypes in women and sheep carrying *BMP15* mutations emphasize the importance of the sheep model to further determine the involvement of BMP15 in ovarian physiology and pathophysiology in women.

## Materials and Methods

### Animals

The French Grivette population is a local breed mainly located in Rhône and Loire French departments. Ewes present naturally good maternal characteristics and a high prolificacy with more than 2 newborns per dam and per year. The very high variability of LS among related ewes suggested the segregation of a major gene affecting this trait. To test this assumption, 29 highly prolific ewes (LS≥2.7) considered as cases and 11 normally prolific ewes (LS≤1.8) considered as controls were selected to perform a genome-wide association study (GWAS) using a case/control design.

The Polish Olkuska population is a native Polish long-wool breed, traditionally kept in the southern region of the country, near Olkusz and Cracow. The Olkuska is an endangered prolific breed which in 90thies comprised only about 100 ewes dispatched in a few flocks, two of them belonging to Cracow and Warsaw Agricultural Universities that ensured the survival of this breed (reviewed in [36]). At present, due to a successful conservation program, the population increased to over 800 ewes. The main feature of the breed is its exceptional prolificacy, well over 200%, with also a very large variability of LS and OR for related animals. A wide range of mean individual OR (from 1.33 on 9 records to 6.20 on 15 records) led to assume the segregation of a putative major gene increasing OR. 29 cases ewes with high repeated OR (μ≥3.00) and 35 controls ewes with normal OR (μ≤2.00) were chosen to conduct a GWAS. Ovulation rates have been determined by laparoscopy. The laparoscopy was performed using a 5 mm diameter Vega S endoscope by experienced operators.

The description of the study design is presented in Table S1.

To confirm the effect of mutations on LS and OR in both Grivette and Olkuska populations, additional individuals have been genotyped and phenotyped. In total, 360 Grivette ewes coming from 9 flocks for which LS had been measured at least twice have been analyzed. In addition, laparoscopies performed at least 3 times have been obtained on 27 Grivette ewes in order to validate the effect of the mutation on OR. For the Olkuska population, the effect of the mutation on OR has been estimated from 103 ewes checked by laparoscopy (n>3) and LS data (n>2) have been retrieved for 137 animals.

### Samples

Jugular venous blood samples were collected by venipuncture. Genomic DNA was extracted from blood samples following a salt-based DNA extraction as described in [15]. For the Grivette population, all procedures were approved by the “Direction Départementale des Services Vétérinaires de Haute-Garonne” (approval number C31-429-01) for the agricultural and scientific research agency INRA (French National Institute for Agricultural Research), and conducted in accordance with the Guide for the Care and Use of Agricultural Animals in Research and Teaching. For the Olkuska population, all procedures were performed with permission of the animal welfare commission from the University.

### Genotyping analyses

Genotyping for the whole genome scan was performed on the Illumina OvineSNP50 Genotyping Beadchip according to the manufacturer's protocol (http://www.illumina.com). Individuals with a Call Rate <0.98 were excluded. SNPs were excluded if they showed a Call Freq <0.98, a minor allele frequency (MAF) <0.01 in cases or controls or significant deviation from Hardy-Weinberg equilibrium (HWE) in the controls (p<1E^−06^). Non polymorphic SNPs and markers with no position on the OARv2.0 map (http://www.livestockgenomics.csiro.au/sheep/) were also eliminated. Finally, an initial design of 54241 SNPs available on the Illumina OvineSNP50 Genotyping Beadchip and 104 individuals (40 and 64 ewes of the French Grivette and Polish Olkuska protocols respectively) was reduced to final datasets of 47290 SNPs analysed in 39 individuals for the French Grivette population and 46451 SNPs analysed in 63 individuals for the Polish Olkuska population for further analyses as showed in Table S1.

Genotyping of both *FecX^Gr^* and *FecX^O^* mutations was performed by allele-specific amplification using the KASPar SNP genotyping system and followed by fluorescence detection on a ABI7900HT. KASPar assays were carried out in 5 µL reactions according to the KBioscience published conditions (http://www.kbioscience.co.uk/).

The primers used in this study are listed in Table S3.

### Haplotype reconstruction analyses

The most likely linkage phase for each individual was determined using a coalescent theory approach under a Bayesian population genetic model implemented in the PHASE program [51], [52].

### DNA sequencing analyses

Long-range PCR amplifications were performed using the Long PCR Enzyme Mix provided by Fermentas (http://www.fermentas.de) and internal primers were used for the sequencing reaction realized *via* the BigDye Terminator v3.1 Cycle Sequencing Kit (http://www.appliedbiosystems.com).

The primers used in this study are listed in Table S3.

### Construction of BMP15 expression plasmids

BMP15 gene ovine variants (*FecX^Gr^/oBMP15^T317I^* and *FecX^O^/oBMP15^N337H^*) were introduced by site-directed mutagenesis into the pCShBMP15wt vector, containing a full-length human BMP15 wild-type cDNA leading to the *hBMP15^T316I^* and *hBMP15^N336H^*, respectively. For the sake of simplicity, the numbering of ovine BMP15 was kept all along the manuscript. As previously described [32], mutagenesis reaction for each variant was performed using the QuickChange Site-Directed Mutagenesis kit (Stratagene) and specific couples of primers (Table S3).

### Transient transfections and luciferase reporter assay

A COV434 human granulosa cells line stably expressing the BMP responsive element (BRE) - luciferase reporter was transiently transfected with BMP15 expressing vectors as already described by Rossetti et al. [32]. Briefly, COV434 cells were transfected in triplicate with 500 ng/well of pCShBMP15 vector expressing the wild-type or the mutant each alone or in equal combination (250 ng each) by using Fugene HD (Roche Applied Sciences, Indianapolis, IN). Cells were also transfected with the pCS2 empty vector and treated or not by 100 ng/ml of rhBMP-15 (R&D Systems, Minneapolis, MN), as positive or negative control, respectively. Forty hours after transfection, cells were lysed in Passive Lysis Buffer and assayed for luciferase activity using the Dual Luciferase reporter Assay kit (Promega). Luminescence in relative light units (RLU) was measured in a Fluoroskan Ascent instrument (Labsystems, Oy, Finland).

### Statistical analyses

Single-marker association analyses were conducted using a Fisher's exact test and a Bonferroni correction has been applied to check for significance levels. The chromosome-wide and genome-wide values have been established as mentioned by Balding et *al.* [53]. Briefly, Bonferroni correction was applied to both the genome-wide and chromosome-wide analyses. The p-values generated were evaluated according to an adjusted significance threshold generated by dividing the 0.05 threshold by the total number of tests (number of SNPs considered) performed in each case (whole genome or whole chromosome). Statistical analyses were done using the PLINK software [54].

Haplotypic association analysis specifying all haplotypes in sliding windows of a fixed number of SNPs (n = 20) was also performed for X chromosome locus using chi-squared and logistic regression methods similar to other recent approaches [55], [56]. Empirical significance levels were calculated using the maximum statistic permutation approach (max(T), n = 1000).

For genotype effect on OR or LS and reporter luciferase assays, differences between means were analyzed by one-way ANOVA followed by Neuman-Keuls post-hoc test to compare between conditions. p<5E^−02^ was considered statistically significant. All the results were presented as means±SD.

## Supporting Information

Figure S1 Chromatograms of identified *FecX^Gr^* and *FecX^O^* mutations. (A) *BMP15^T317I^* (*FecX^Gr^*) mutation identified in the French Grivette population. (B) *BMP15^N337H^* (*FecX^O^*) mutation identified in the Polish Olkuska population. For each mutation symbolized by an arrow, the 3 genotypes are shown.
(TIF)

Figure S2 Chromosome-wide association results including identified *FecX^Gr^* and *FecX^O^* mutations. (A) Chromosome-wide association results for litter size in the French Grivette sheep population. (B) Chromosome-wide association results for ovulation rate in the Polish Olkuska sheep population. Manhattan plots show the combined association signals (−log_10_(p)) on the y-axis versus SNPs position in the sheep genome on the x-axis and ordered by chromosome number (OARv2.0 available on http://www.livestockgenomics.csiro.au/sheep/ website). Black lines represent the 5% chromosome-wide threshold. Significant association p for *FecX^Gr^* and *FecX^O^* mutations are symbolized by an arrow.
(TIF)

Figure S3 Effect of *FecX^Gr^* and *FecX^O^* mutations on the intrinsic properties of BMP15 protein. (A) Hydrophobicity plots of the mutated and wild type BMP15 proteins based on the hydrophobicity scale described by Kyte et al. [57]. (B) Polarity plots of the mutated and wild type BMP15 proteins based on the polarity scale described by Zimmerman et al. [58]. (C) Molecular weight plots of the mutated and wild type BMP15 proteins. Amino acids from 300 to 350 were represented. Location of both *FecX^Gr^* and *FecX^O^* mutations in the sequence are symbolized by red and green boxes, respectively.
(TIF)

Table S1 Description of the study design. Call Rate is defined as the ratio of number of genotypes exceeding the threshold value to the total number of genotypes. Call Freq is the proportion of all samples at each locus wit call scores above the no-call threshold. Position pb for Position problem corresponds to markers with an identified location in the first sheep map submitted to the Illumina OvineSNP50 Beadchip but unassigned on the OARv2.0 assembly. HWE for Hardy-Weinberg Equilibrium. MAF for Minor Allele Frequency. Values for phenotype are presented as mean (standard deviation).
(TIF)

Table S2 Polymorphisms identified in the BMP15 5′ regulatory region in the Polish Olkuska ewes. Name of the polymorphism corresponds to the location upstream the ATG in the BMP15 sequence.
(TIF)

Table S3 List of primers used in the study. Location of primers are based on the OARv2.0 assembly available on http://www.livestockgenomics.csiro.au/sheep/ website, excepted for primers used for directed mutagenesis based on human BMP15 cDNA (GenBank NM_005448) with mutated nucleotides underlined.
(TIF)
