# Supplementary figures and images for: Genome-Wide Association Studies Identify Two Novel BMP15 Mutations Responsible for an Atypical Hyperprolificacy Phenotype in Sheep
Source: PLoS Genet. 2013 Apr 25;9(4):e1003482. doi: 10.1371/journal.pgen.1003482 (PMC3636084; doi:10.1371/journal.pgen.1003482)

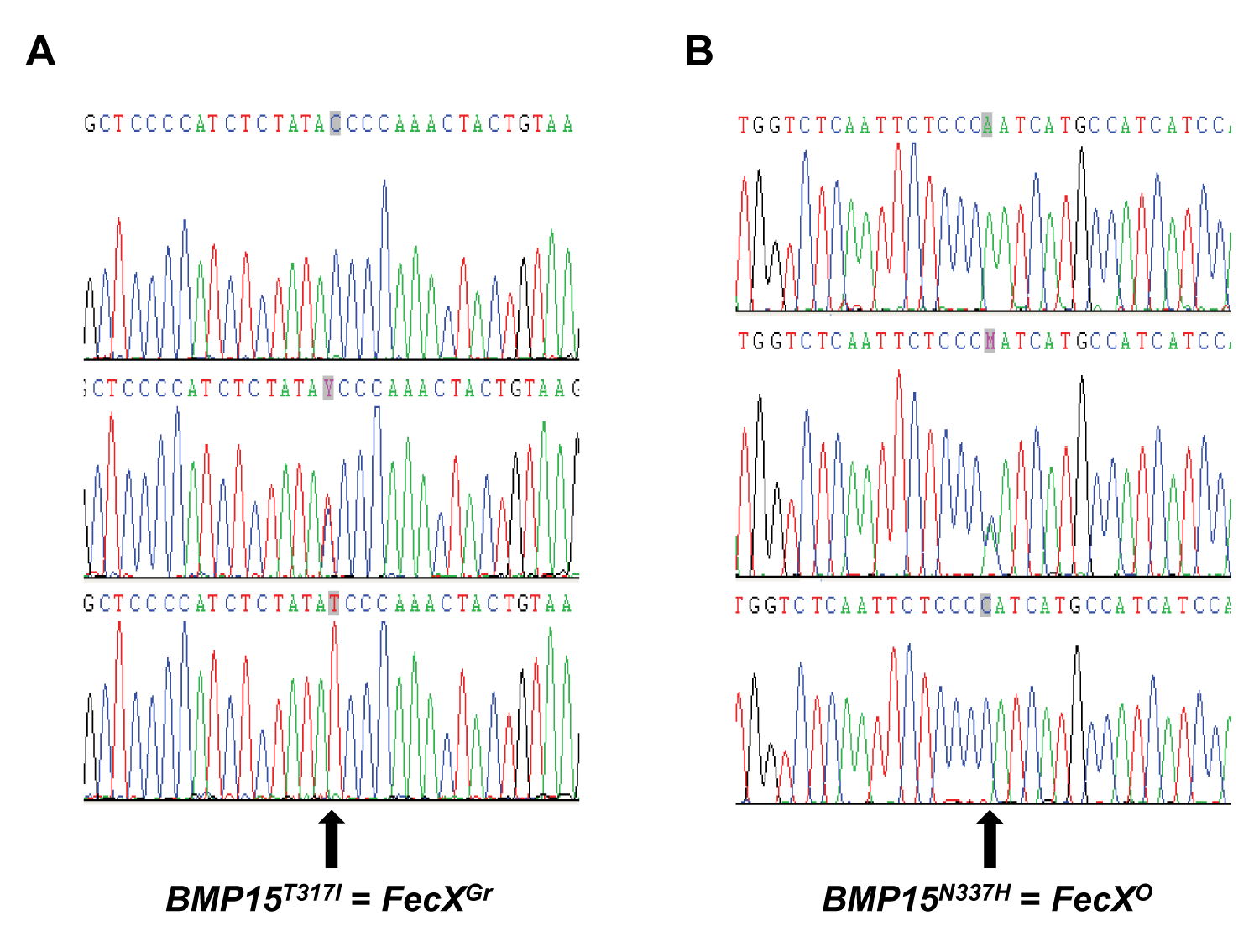

Supplement: Figure S1 — Chromatograms of identified FecXGr and FecXO mutations. (A) BMP15T317I (FecXGr) mutation identified in the French Grivette population. (B) BMP15N337H (FecXO) mutation identified in the Polish Olkuska population. For each mutation symbolized by an arrow, the 3 genotypes are shown. (TIF) [file pgen.1003482.s001.tif]

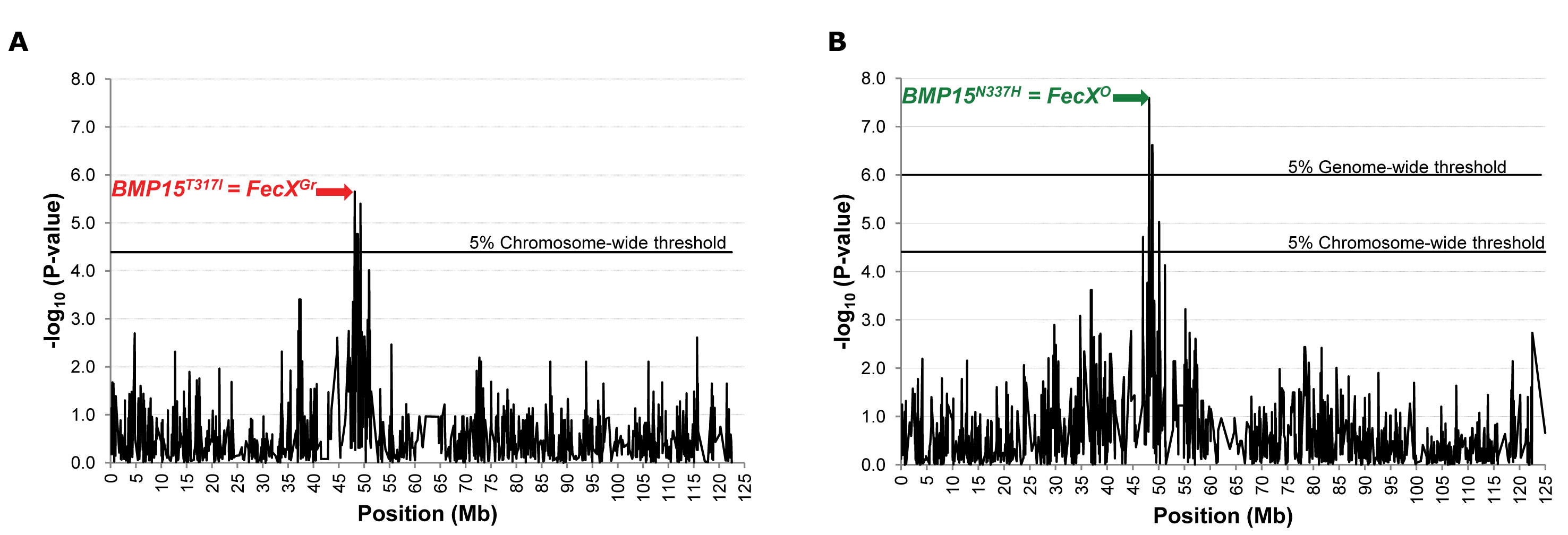

Supplement: Figure S2 — Chromosome-wide association results including identified FecXGr and FecXO mutations. (A) Chromosome-wide association results for litter size in the French Grivette sheep population. (B) Chromosome-wide association results for ovulation rate in the Polish Olkuska sheep population. Manhattan plots show the combined association signals (−log10(p)) on the y-axis versus SNPs position in the sheep genome on the x-axis and ordered by chromosome number (OARv2.0 available on http://www.livestockgenomics.csiro.au/sheep/ website). Black lines represent the 5% chromosome-wide threshold. Significant association p for FecXGr and FecXO mutations are symbolized by an arrow. (TIF) [file pgen.1003482.s002.tif]

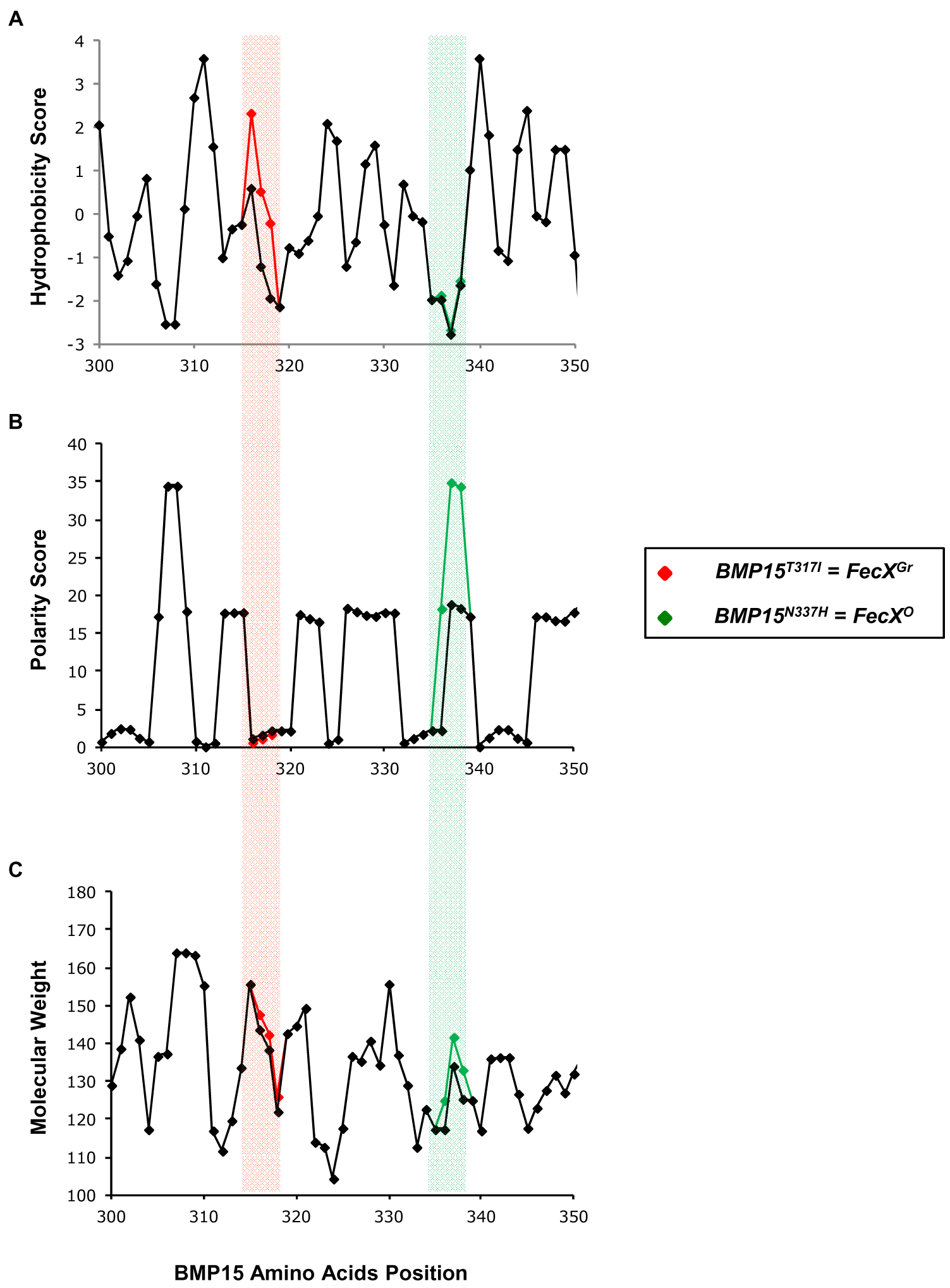

Supplement: Figure S3 — Effect of FecXGr and FecXO mutations on the intrinsic properties of BMP15 protein. (A) Hydrophobicity plots of the mutated and wild type BMP15 proteins based on the hydrophobicity scale described by Kyte et al. [57]. (B) Polarity plots of the mutated and wild type BMP15 proteins based on the polarity scale described by Zimmerman et al. [58]. (C) Molecular weight plots of the mutated and wild type BMP15 proteins. Amino acids from 300 to 350 were represented. Location of both FecXGr and FecXO mutations in the sequence are symbolized by red and green boxes, respectively. (TIF) [file pgen.1003482.s003.tif]

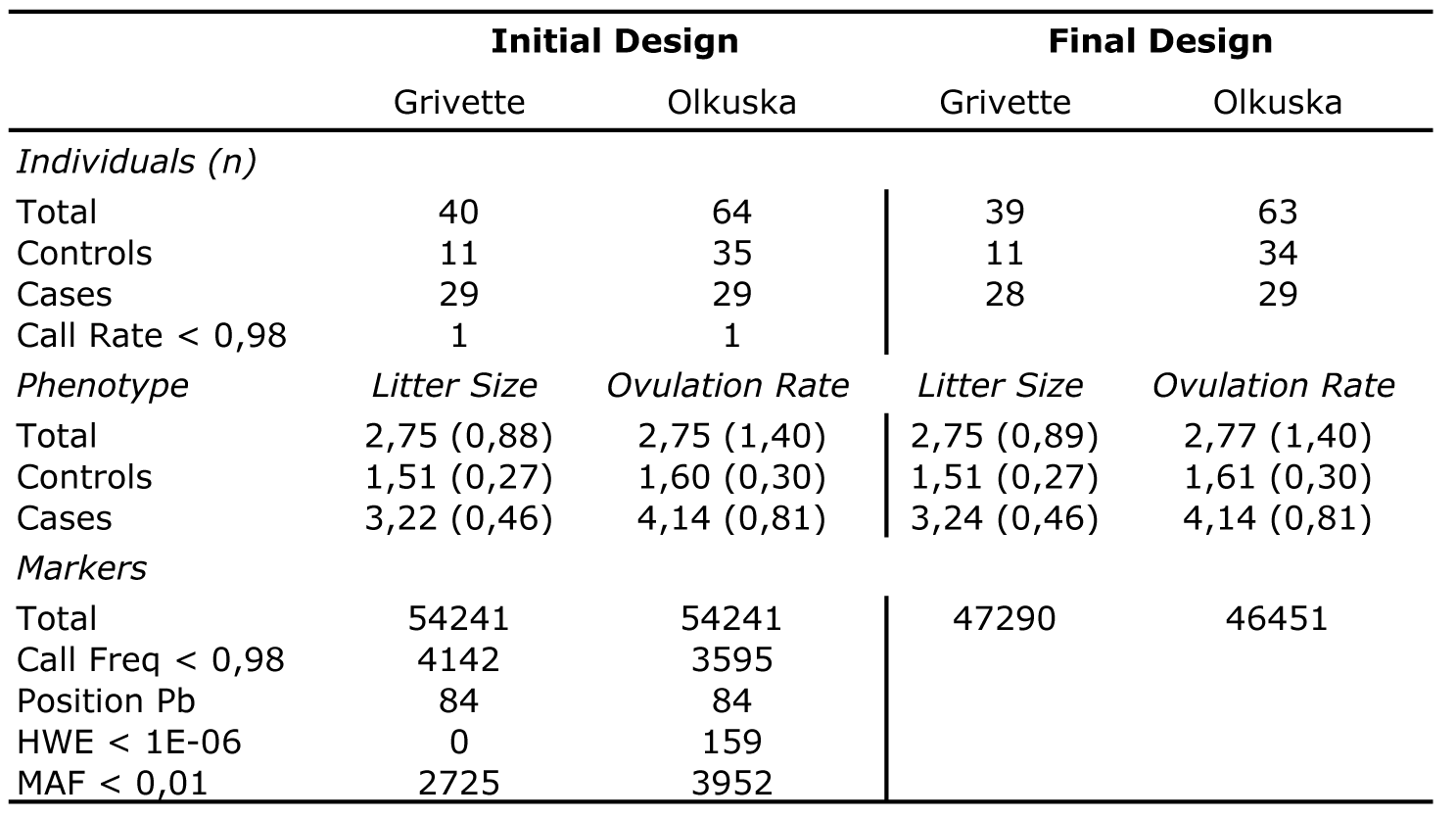

Supplement: Table S1 — Description of the study design. Call Rate is defined as the ratio of number of genotypes exceeding the threshold value to the total number of genotypes. Call Freq is the proportion of all samples at each locus wit call scores above the no-call threshold. Position pb for Position problem corresponds to markers with an identified location in the first sheep map submitted to the Illumina OvineSNP50 Beadchip but unassigned on the OARv2.0 assembly. HWE for Hardy-Weinberg Equilibrium. MAF for Minor Allele Frequency. Values for phenotype are presented as mean (standard deviation). (TIF) [file pgen.1003482.s004.tif]

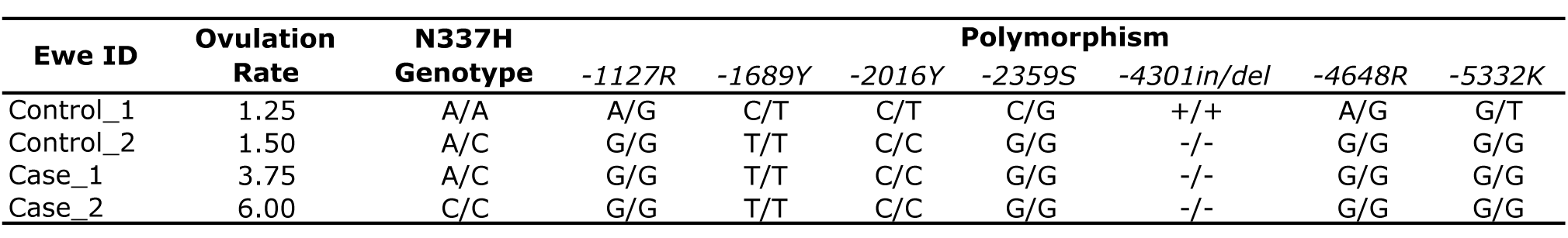

Supplement: Table S2 — Polymorphisms identified in the BMP15 5′ regulatory region in the Polish Olkuska ewes. Name of the polymorphism corresponds to the location upstream the ATG in the BMP15 sequence. (TIF) [file pgen.1003482.s005.tif]

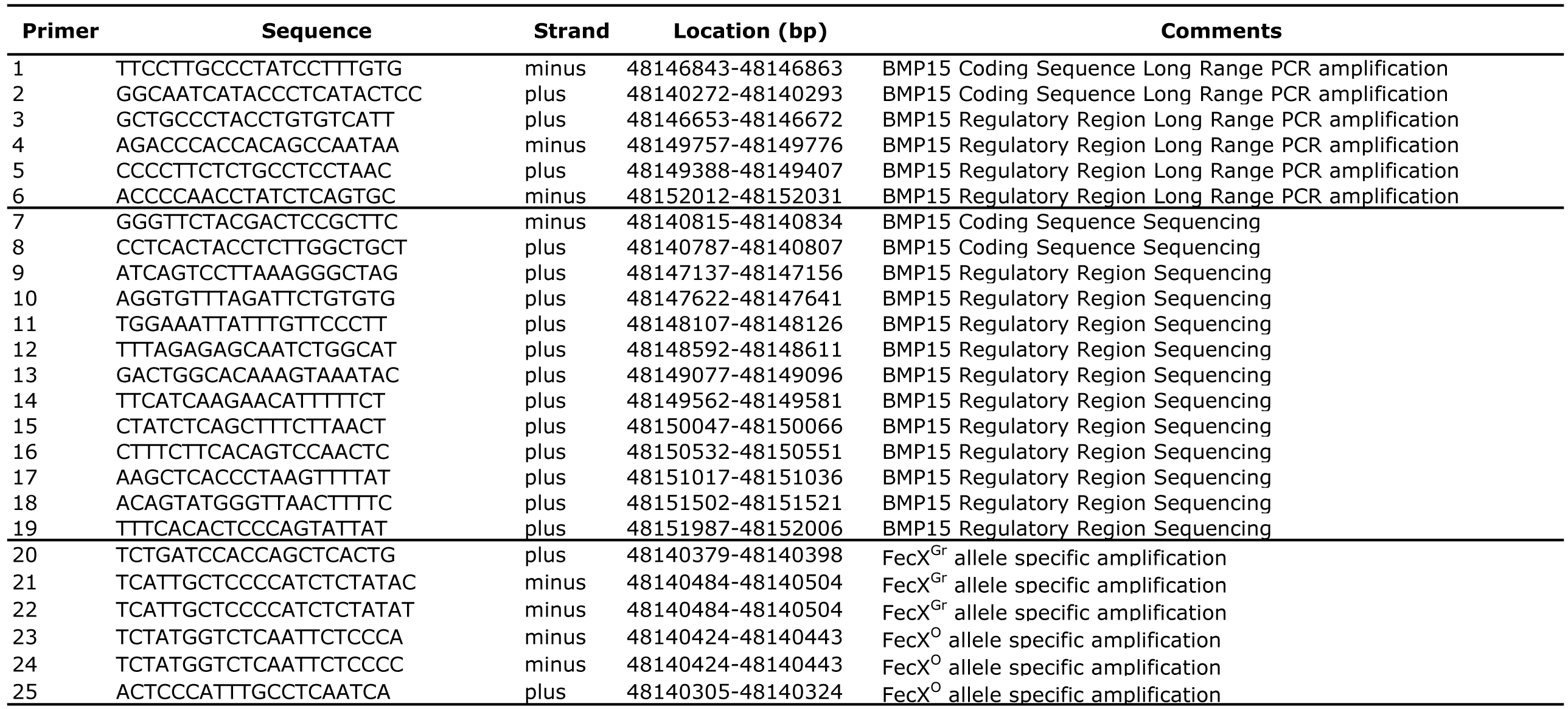

Supplement: Table S3 — List of primers used in the study. Location of primers are based on the OARv2.0 assembly available on http://www.livestockgenomics.csiro.au/sheep/ website, excepted for primers used for directed mutagenesis based on human BMP15 cDNA (GenBank NM_005448) with mutated nucleotides underlined. (TIF) [file pgen.1003482.s006.tif]
